# Editorial for the Special Issue of Selected Papers from the 8th Symposium on Micro–Nano Science and Technology on Micromachines

**DOI:** 10.3390/mi9120627

**Published:** 2018-11-28

**Authors:** Norihisa Miki, Koji Miyazaki, Yuya Morimoto

**Affiliations:** 1 Department of Mechanical Engineering, Keio University, 3-14-1 Hiyoshi, Kohoku-ku, Yokohama, Kanagawa 223-8522, Japan; 2 Department of Mechanical and Control Engineering, Kyushu Institute of Technology, 1-1 Sensui-cho, Tobata-ku, Kitakyushu, Fukuoka 804-8550, Japan; miyazaki@mech.kyutech.ac.jp; 3 Institute of Industrial Science (IIS), The University of Tokyo, 4-6-1 Komaba, Meguro-ku, Tokyo 153-8505, Japan; y-morimo@iis.u-tokyo.ac.jp

The Micro–Nano Science and Technology Division of JSME (Japan Society of Mechanical Engineers) promotes academic activities to pioneer novel research topics on microscopic mechanics. The division encourages interdisciplinary studies to more deeply understand physical/chemical/biological phenomena on the micro/nano scale and to develop applied technologies. Since 2009, seven symposiums on Micro–Nano Science and Technology have taken place in a more interdisciplinary manner, incorporating the related societies of electronics and applied physics. We have promoted in-depth studies and interactions between researchers/engineers in various fields with more than 140 papers presented at each symposium over the past years. Thanks to these previous activities and the great effort of the committee members, the Micro–Nano Science and Technology Division has been recognized as a formal division of JSME.

This Special Issue collects 13 papers from the 8th Symposium on Micro–Nano Science and Technology, which was held from 31 October to 2 November, 2017 in Hiroshima, Japan. All of the papers highlight new findings and technologies at the micro/nano scales relating to a wide variety of fields of mechanical engineering, from fundamentals to applications. 

Micro/nano fluidics have been studied using both fundamental and application-driven approaches. The visualization of the pH distribution around an ion depletion zone in a microchannel was successfully presented [1]. This technique and the knowledge that can be obtained by it will be indispensable for designing effective nano-channels for bio/chemical applications. Microfibers that can encapsulate cells and microbes will be a useful tool for fundamental biology, such as cell characterization and biomedical and environmental applications. The formation of branched and chained alginate microfibers was presented [2]. Considering the practical applications of bioremediation, a triple-coaxial flow device for the mass-production of hydrogel micro tubes containing microbes was designed and fabricated [3]. 

Materials and manufacturing technologies have always formed the core of micro/nano science and technologies. The crack-configuration of metal conductive tracks embedded in stretchable elastomers was analyzed thoroughly in [4], contributing to flexible electronics. The 3D shape reconstruction of 3D-printed transparent microscopic objects was demonstrated to further expand the design spaces for micro/nano structures [5]. The reductive sintering of mixed CuO/NiO nanoparticles using a femtosecond laser was intensively characterized, particularly with respect to heat accumulation [6]. The formation of arbitrary 3D shapes is a great challenge for micro/nano objects. Origami-like folding deformation [7] and self-organization using cellular automata [8] were successfully demonstrated.

Physical sensors are a major application of micro/nano technologies. A biomimetic, artificial, sensory epithelium was designed and demonstrated [9]. Micro cantilever arrays work as the tactile sensor for gripping control in [10]. Simple methods for the reduction of the parasitic capacitance of a flexible polymer-based capacitive senor was proposed in [11].

The applications enabled by micro/nano technology-based sensors include human fatigue assessment [12] and adenosine triphosphate measurement in deep seas [13]. Such research requires the integration of the technologies of sensors, systems, and experiments. Micro/nano research mainly focuses on the phenomena and technologies at micro/nano scales. However, in order to make micro/nano research applicable, macroscopic viewing and technologies must be incorporated. 

We would like to thank all the contributing authors for their excellent research work. We appreciate all the reviewers who provided valuable comments to improve the quality of the papers and the tremendous support from the editorial staff of *Micromachines*.
